# 

**Published:** 1954-10

**Authors:** 


### THE MEDICAL JOURNAL OF THE SOUTH-WEST

*Incorporating*
*The Bristol Medico-Chirurgical Journal*

ARMED FORCES
FEB 15 1955
MEDICAL LIBRARY

*October 1954*

VOL. 70 (iv). No. 256 Price 4/-

